# Supplementary material for: Using 3D Invasion properties of RCC Cell Lines In Vitro to predict their Metastatic Potential In Vivo
Source: Cell Death Discov. 2026 Feb 27;12:122. doi: 10.1038/s41420-026-02966-7 (PMC13031655; doi:10.1038/s41420-026-02966-7)
Supplement: Supplementary file 8 — Uncropped western blots [file 41420_2026_2966_MOESM8_ESM.pdf]

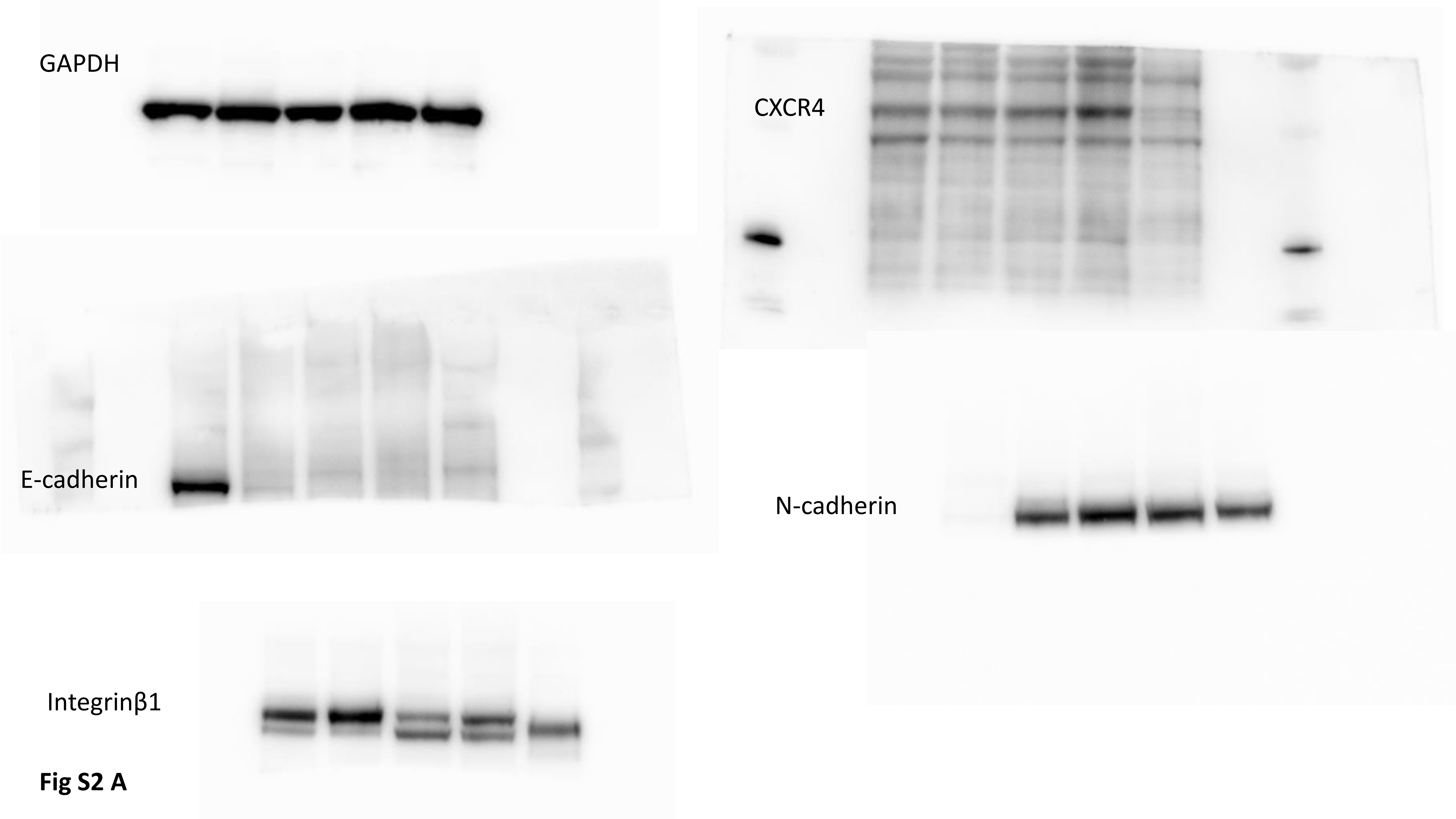

Fig S2 A

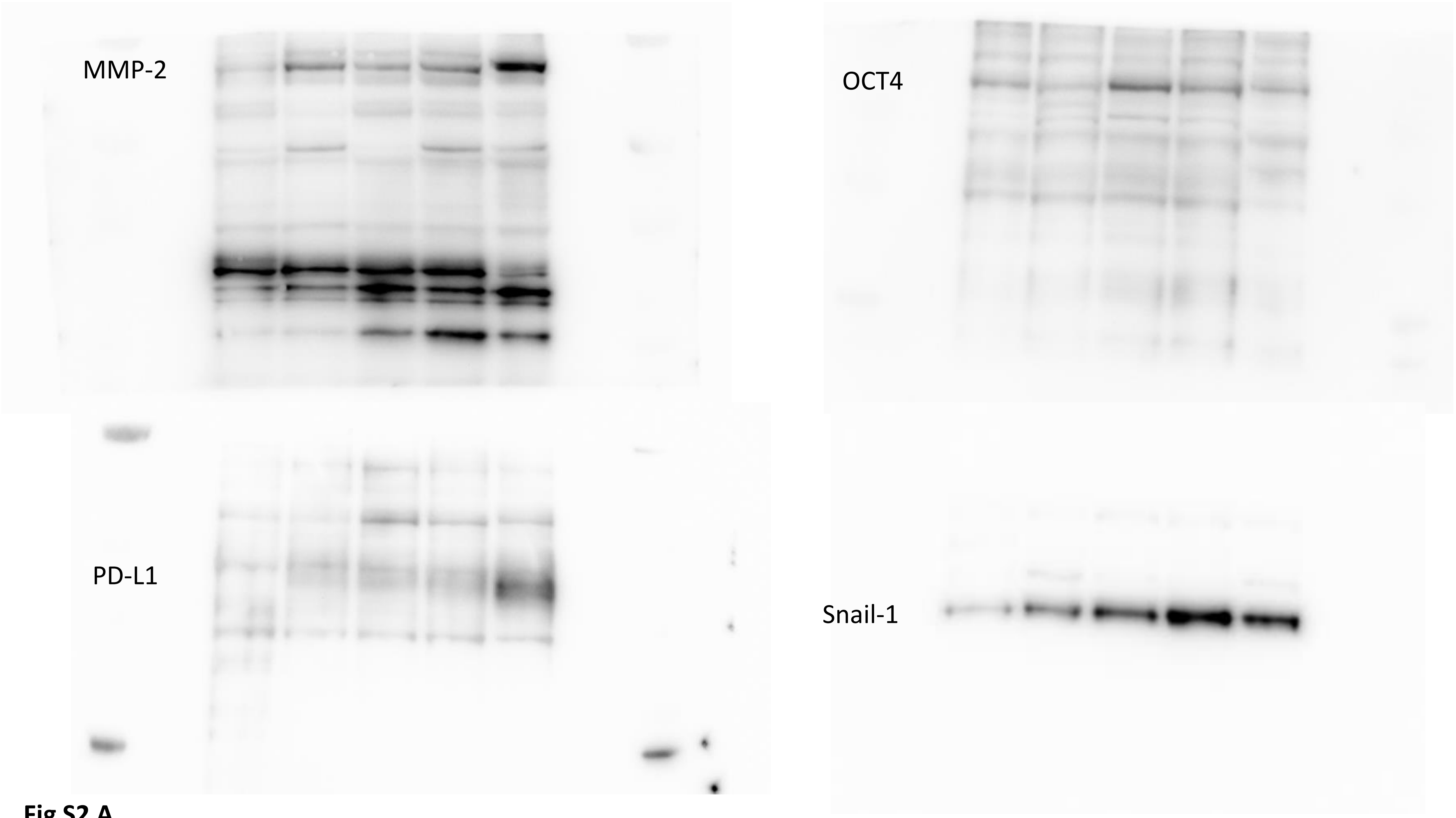

Fig S2 A

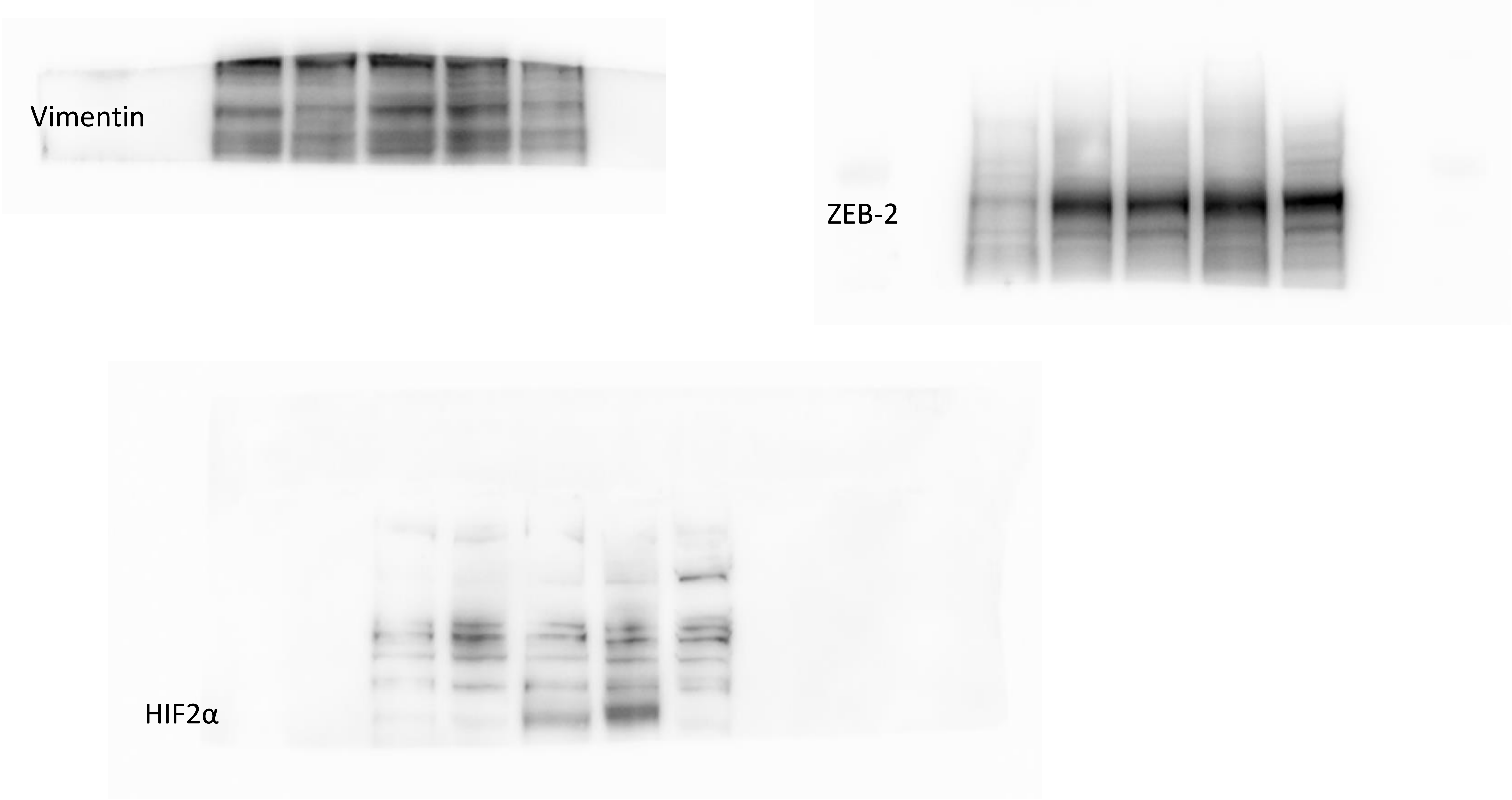

Fig S2 A

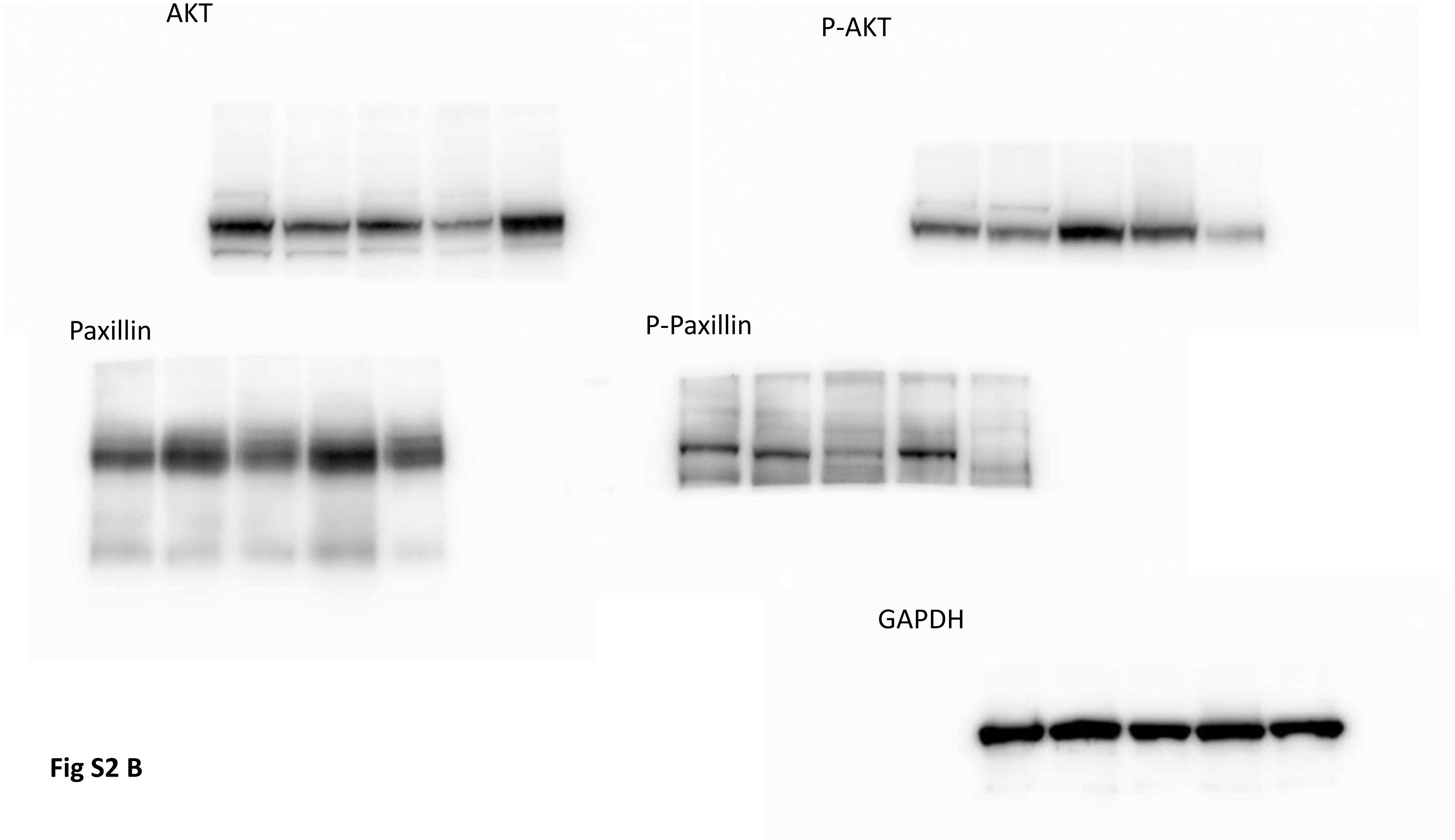

Fig S2 B
