# Supplementary material for: Using 3D Invasion properties of RCC Cell Lines In Vitro to predict their Metastatic Potential In Vivo
Source: Cell Death Discov. 2026 Feb 27;12:122. doi: 10.1038/s41420-026-02966-7 (PMC13031655; doi:10.1038/s41420-026-02966-7)
Supplement: Supplementary file 9 — Supplementary Material and Methods [file 41420_2026_2966_MOESM9_ESM.pdf]

Supplementary Material and Methods

Preparation of cell extracts

RPTEC, ACHN, RCC10, 786-O and RCC7 cells were cultured to reach sub-confluency. Cells were washed with PBS and frozen at - 80°C. Cells were lysed on ice for 30 min in RIPA buffer (10 mM Tris-HCL, pH 7.4, 150 mM NaCl, 1% Triton X-100, 0.1% SDS, 0.5 % DOC, 1 mM EDTA) with protease and phosphatase inhibitor cocktail (Sigma Aldrich, P8340, P2850, P5726) at the recommended concentrations, centrifuged for 15 min at 4°C at 13.000 rpm and the supernatants collected. Proteins were quantified using the BCA protein assay kit (Pierce, ThermoFisher Scientific).

Immunoblotting

Antibodies used for western blot analysis included GAPDH (#AM4300) from Invitrogen. HIF2α (#100-122) from Novus Biologicals. E-Cadherin (#610404), N-Cadherin (#610920), β1 Integrin (#610467 from BD Transduction Laboratories. Vimentin (#V5255), ZEB2 (#C83384) from Sigma Aldrich. PDL-1 (#13684), OCT4 (#2750), Snail1 (#3895), MMP2 (#4022), AKT (#9272), P-AKT S473 (#4060), P-Paxillin1 Y118 (#2541) from Cell Signaling Technology. Paxillin1 (#610052) from BD Biosciences. CXCR4 (#ab124824) from Abcam. After three washes, secondary antibodies (peroxidase-conjugated affinity pure anti-rabbit IgG (#111035OO3) or goat anti-mouse IgG (#115035003) from Jackson Immuno Research) were applied for 1 h followed by three more washes with TBST. Immobilon Forte Western HRP substrates (Millipore) was added and detection was achieved by using a Fusion FX acquisition system (Vilbert). Anti-GAPDH was used as a loading control and band intensities were quantified using ImageJ.

2D cell assays proliferation

786-O, RCC10, and RCC7 cells were seeded in 96-well plates (5,000/well). Cells were monitored for 5 days with CELLCYTE X™ ECHO (10X, every 2 h), cell proliferation was tracked in real time, and confluence over time (%/h) was analyzed via image software (Cellcyte Live Cell Analyzer software).

2D cell migration

786-O, RCC7, and RCC10 cells were seeded in 96-well plates (30,000 cells/well). After 24 h, mitomycin C was applied for 2 h to inhibit proliferation [16], and an 800 µm wound was created using WoundMaker (Essen Biosciences). Cells were monitored for 3 days with CELLCYTE X™ ECHO (10X, every 2 h), and confluence was analyzed over time (%/h).

2D cell invasion

786-O, RCC7 and RCC10 cells were seeded (200,000) cells in Matrigel-coated Boyden chambers (Corning® BioCoat Matrigel) with a 2 %-20 % FBS serum gradient. After 48 h at 37°C, cells were fixed in 4% PFA for 15 min and stained with Hoechst 33342 (1 µg/ml). Non-invasive cells were removed and membraned were mounted and observed (AxioObserver Z1, Zeiss, TILES mode, 10X). Invading cells were quantified using ImageJ.

Immunofluorescent labeling of tumoroids

Tumoroids were washed with PBS, fixed using 4% paraformaldehyde (PFA) (Sigma, 1 h, 4°C). All subsequent steps were carried out with gentle agitation (60 rpm). Following a 10 min PBS-Tween20 wash, samples were permeabilized and blocked for 1 h in Organoid Washing Buffer (OWB), containing 0.1% Triton-X100 and 0.2% BSA in PBS. Primary antibodies were incubated overnight (4°C), followed by 3 OWB washes (2 h) and secondary antibodies incubation overnight (4°C). On the third day, tumoroids underwent 3 additional OWB washes, with Hoechst (1 µg/ml) included in the second wash for nuclear staining. The primary antibodies used were CA9 (NovusBio, #NB100-417) and Phalloidin (Invitrogen, #A12379). The secondary antibody applied was Cy3 Goat anti-rabbit IgG (Jackson ImmunoResearch, #111-165-003). Imaging was performed using a Zeiss Apotome microscope.

Image analysis

To measure the average velocity of proliferation and wound closure over the measured time points (%/h), the confluence values were first normalized to account for variations in initial confluence across different cell types and replicates. The velocity was then calculated by performing linear regression analysis on the normalized confluence values.

Transcriptomic analysis

RNA extracts from spheroids (786-O, RCC7 and RCC10) were obtained using the RNeasy Micro Kit (Qiagen) following manufacturer's instructions. Five spheroids were used for each extract. High sensitivity Bulk RNA Barcoding (BRB)-sequencing and raw data preprocessing were performed by Alithea Genomics | MERCURIUS™ High Throughput Transcriptomics Service. Subsequent analysis was realized in R version 4.2.1. Gene expression is expressed in log scale, and comparison between cell types are performed with visual inspection and Pearson correlation coefficient. The EMT genes are taken from the Molecular Signatures Database (MSigDB) gene sets (library msigdbr in R version 7.5.1). EMT score is defined as the average of the log2 scale gene expression of the EMT genes per cell line.
