# Supplementary material for: Using 3D Invasion properties of RCC Cell Lines In Vitro to predict their Metastatic Potential In Vivo
Source: Cell Death Discov. 2026 Feb 27;12:122. doi: 10.1038/s41420-026-02966-7 (PMC13031655; doi:10.1038/s41420-026-02966-7)
Supplement: Supplementary file 10 — S1; S2; S3; S4 and S5 [file 41420_2026_2966_MOESM10_ESM.pdf]

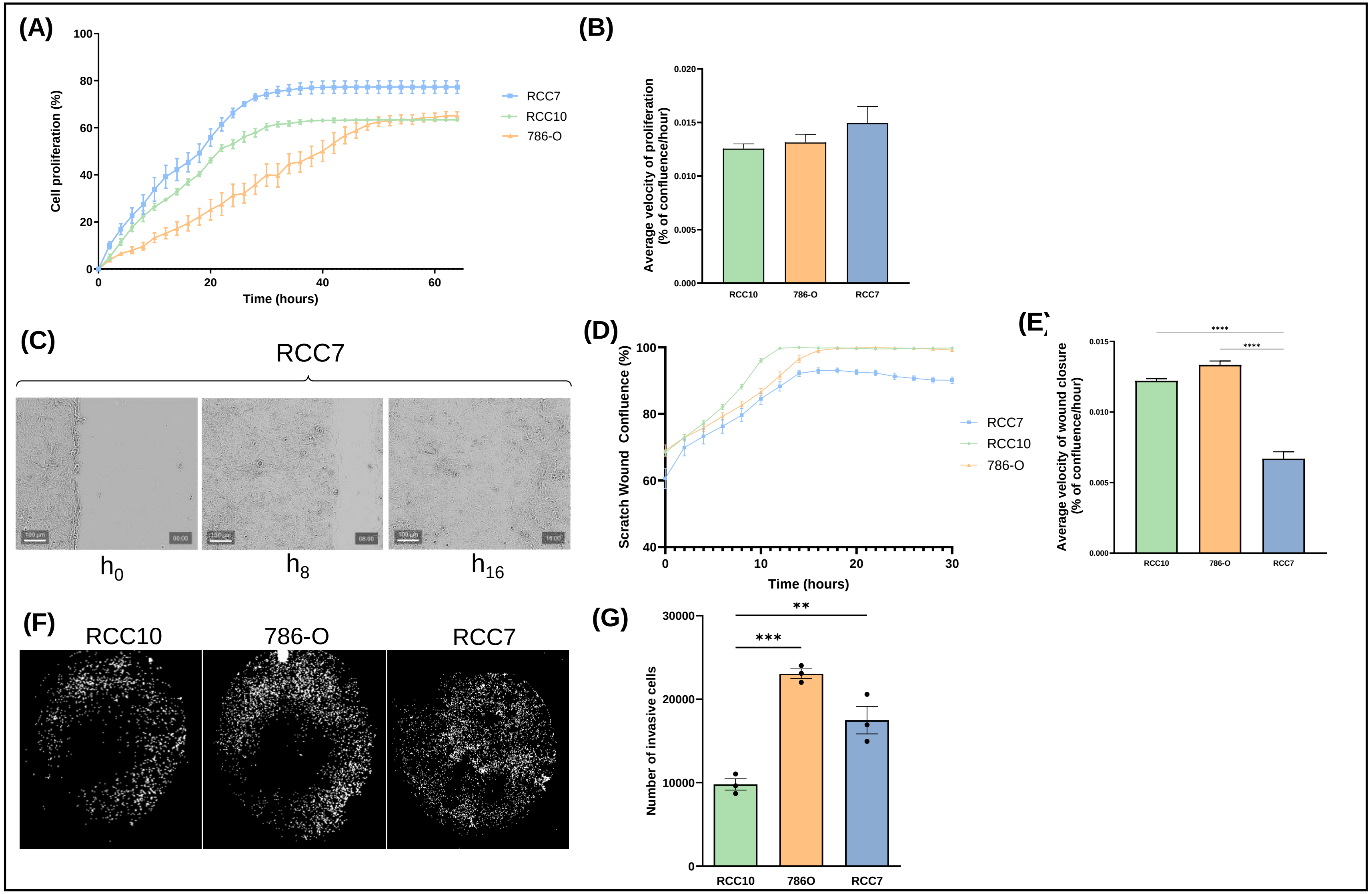

Fig S1 : 2D Cell line-specific proliferation and migration (A) Proliferation assay of RCC cell lines performed with CELLCYTE X™ Live Cell Imager And Analyzer **(B)** Average velocity of proliferation (% of confluence/hour) **(C)** Representation of the Scratch Wound Healing Assay for the RCC7 cell line **(D)** Scratch wound confluence data (%) for all RCC cells after 30 hours of culture, performed with CELLCYTE X™ Live Cell Imager. **(E)** Average velocity of wound closure (%of confluence/hour) **(F)** Image representation of the membranes from the Matrigel-coated Boyden invasion assays with indicated RCC cell lines. Cell nuclei are stained with Hoechst 33342. **(G)** Number of invasive cells counted on the bottom of each Matrigel-coated Boyden membrane. Data show mean ± SEM, with n = 6 for the proliferation assay, n = 12 for the Scratch Wound Healing Assay and n = 3 for the Matrigel-coated Boyden invasion assay. Significance was assessed using a one-way ANOVA with Tukey's multiple comparison test comparing each cell line to every other cell line. (***p*<0,01 ; ****p*<0,001 ; **** *p*<0,0001)

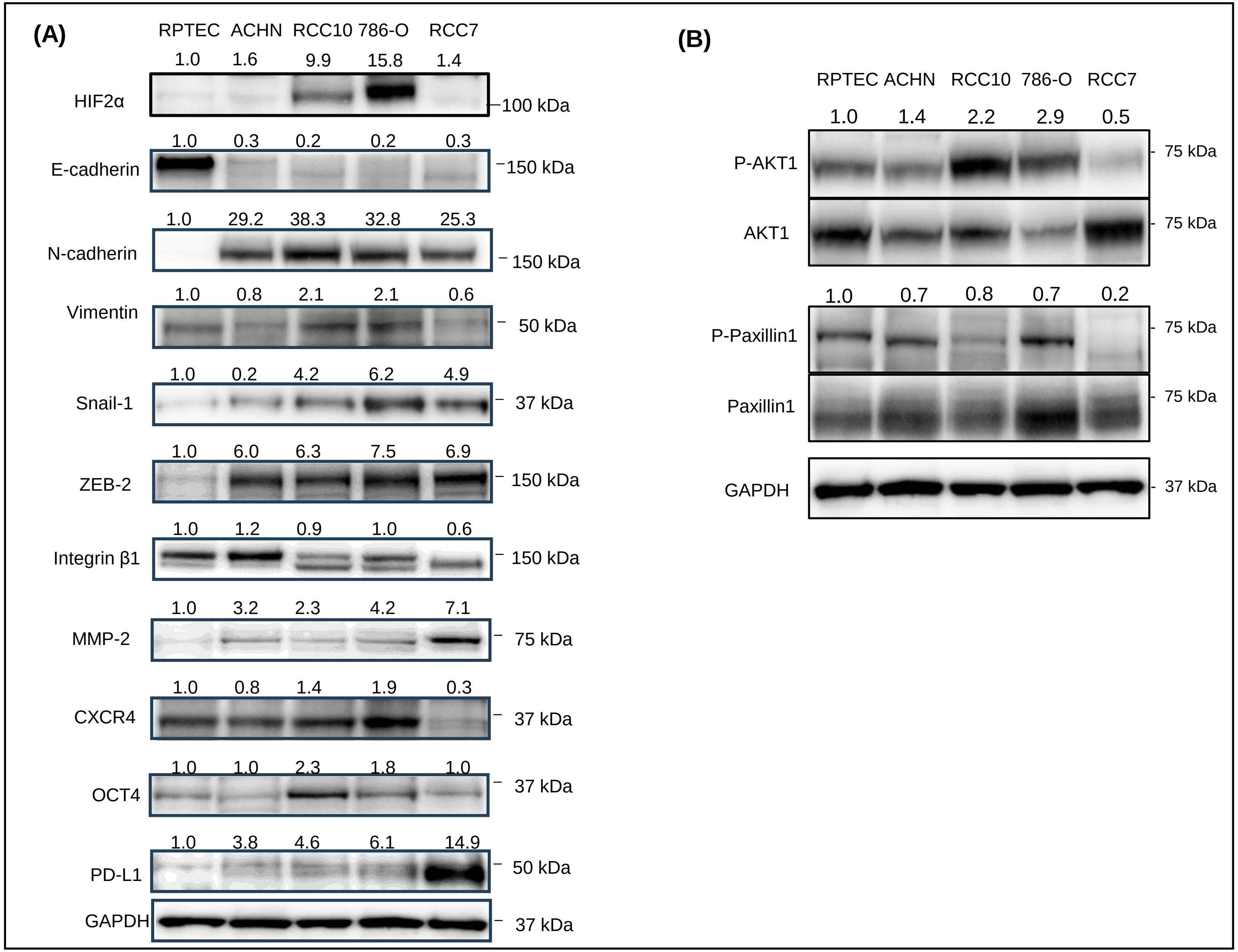

Fig S2 : Western Blot analysis of key signaling pathways in RCC cell lines and in RPTEC, a healthy cell line. Representative Western Blot analysis comparing proteins involved in the EMT (**A**) and phosphorylation pattern of AKT1 and Paxillin1 **(B).** GADPH was used for normalization of protein loading. Quantification of band intensities from the Western blot A and the ratio of the phosphorylated protein over the total protein B, are indicated above each lane.

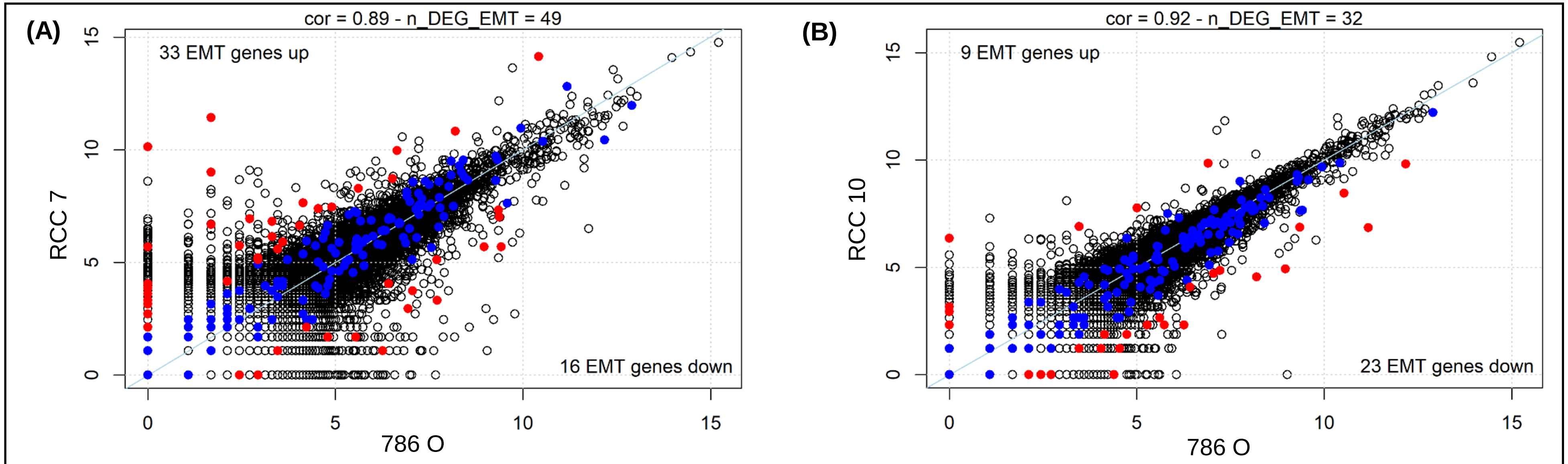

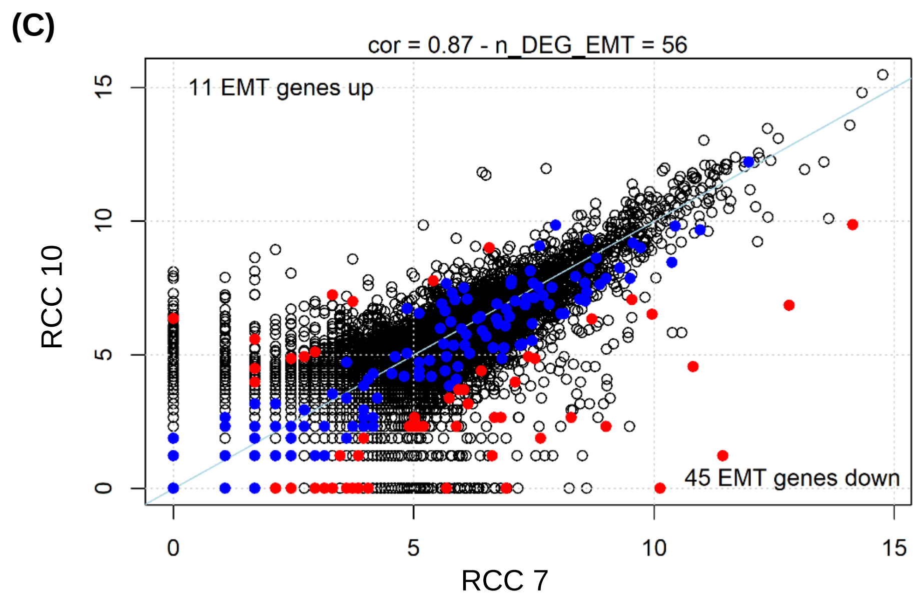

(D)

Cell Line	EMT Score
RCC10	4.23
786-O	4.63
RCC7	5.16

Fig S3 : 3D Cell line transcriptome and expression of the EMT genes. (A-C) Comparison of gene expression in log2 scale for RCC7, 786-O and RCC10 cell lines grown in 3D. Pearson correlation coefficient and the number of EMT differentially expressed genes (DEG_EMT, defined as four times more expressed in one condition versus the other are indicated on top of the plot. The 194 EMT genes are indicated in color, red if the differential expression if more than 4 folds (a log2 scale difference of 2). The number of over-expressed genes in one condition is indicated top-left or bottom-right. **(A)** Gene expression of RCC7 as a function of 786-O. **(B)** Gene expression of RCC10 as a function of 786-O. **(C)** Gene expression of RCC10 as a function of RCC7. **(D)** EMT score define as the average of the log2 scale gene expression of the EMT genes per cell line.

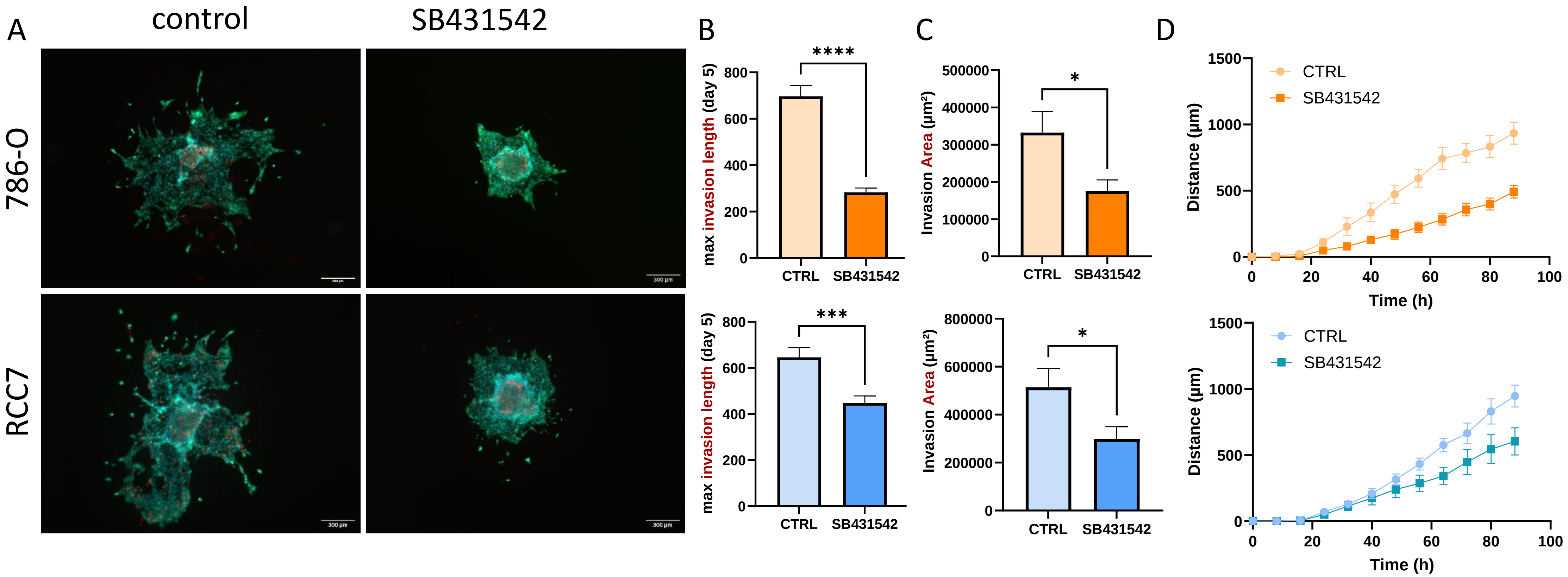

Fig S4 : Inhibition of TGFβ signaling reduces invasive potential of RCC spheroids. (A) Representative images of RCC7 and 786-O spheroids cultured in Collagen I and Fibronectin hydrogel, treated with 5 µM of SB431542 versus vehicle (CTRL), visualized after 5 days with Live and Dead staining (Calcein staining living cells in green, Hoechst 33342 staining nuclei in blue and Ethidium Homodimer I staining dead cells in red). Scale bar = 300 µm. **(B)** Maximum invasion length of RCC cell lines invading after 5 days of culture. **(C)** Average invasion area of ccRCC cell lines invading after 5 days of culture. **(D)** Invasion speed of RCC7, 786-O and RCC10 spheroids embedded in hydrogel measured during 5 days. Data show mean ± SEM, RCC7 n = 8, 786-O n = 8. Significance was assessed using a one-way ANOVA with Tukey's multiple comparison test comparing each cell line to every other cell line. (*p<0,05 ; *** p<0,001 ;**** p<0,0001)

(A)

Mean Cq Alu Seq in lower CAM			
Samples	RCC10	786-O	RCC-7
1	25,73	25,095	24,625
2	23,535	20,905	23,985
3	24,19	23,08	23,805
4	24,23	24,11	24,005
5	24,41	21,67	23,415
6	24,285	24,14	23,72
7	24,315	23,995	23,81
8			23,425

Mean Cq ALU seq CTRL	24,405
SD Alu seq CTRL	0,2
Background threshold	24,205

(B)

Mean Cq Alu Seq in lungs			
Samples	RCC10	786-O	RCC-7
1	28,14	22,06	27,14
2	23,93	26,96	25,89
3	27,01	24,00	25,95
4	26,71	27,55	26,50
5	27,35	26,47	26,67
6	27,48	26,99	24,89
7	24,90	27,04	26,61
8	27,34	27,16	27,54

Mean Cq Alu seq CTRL	26,71
SD Cq Alu seq CTRL	0,33
Background threshold	26,37

(C)

Target Gene	Forward (5'-3')	Reverse (5'-3')	Probe (5'-3')
Alu sequences	GGTGAAACCCCGTCTCTACT	GGTTCAAGCGATTCTCCTGC	CGCCCGGCTAATTTTTGTAT

(D)

Gr. #	Group Description	Total	Alive	Dead	% Alive	% Dead
1	RCC10	17	16	1	94.12	5.88
2	786-O	16	16	0	100	0
3	RCC7	20	16	4	80	20

Fig S5 : Quantitative Evaluation of Metastasis. (A) Duplicates mean value of quantification cycles (Cq) measured by qPCR for human specific Alu sequences (Alu Seq) in the lower CAM. Green values show positive signal, determined by a mean Cq value higher than the background threshold of the qPCR corresponding with the mean value of Cq of control (CTRL) samples minus its standard deviation (SD). **(B)** Duplicates mean value of quantification cycles (Cq) measured by qPCR for human specific Alu sequences (Alu Seq) in the chicken lungs. Green values show positive signal. **(C)** Nucleotide sequences of forward, reverse and probe primers used for the detection of Alu sequences by qPCR based on the protocol described by Funakoshi & al [14]. **(D)** Number of dead and surviving embryos per group.
